# Effects of Different Levels of Pomegranate Seed Oil on Some Blood Parameters and Disease Resistance Against *Yersinia ruckeri* in Rainbow Trout

**DOI:** 10.3389/fphys.2018.00596

**Published:** 2018-05-23

**Authors:** Ümit Acar, Vincenzo Parrino, Osman Sabri Kesbiç, Giuseppe Lo Paro, Concetta Saoca, Francesco Abbate, Sevdan Yılmaz, Francesco Fazio

**Affiliations:** ^1^Bayramic Vocational School, Çanakkale Onsekiz Mart University, Çanakkale, Turkey; ^2^Department of Chemical, Biological, Pharmaceutical, and Environmental Sciences, University of Messina, Messina, Italy; ^3^Faculty of Veterinary, Kastamonu University, Kastamonu, Turkey; ^4^Department of Veterinary Sciences, University of Messina, Messina, Italy; ^5^Department of Aquaculture, Faculty of Marine Sciences and Technology, Çanakkale Onsekiz Mart University, Çanakkale, Turkey

**Keywords:** herbal feed additives, growth performance, *Oncorhynchus mykiss*, *Punica granatum*, sustainable aquaculture

## Abstract

This study is aimed to assess the effects of pomegranate seed oil (PSO) supplementation on growth performance, some hematological, biochemical and immunological parameters, and disease resistance against *Yersinia ruckeri* in cultured rainbow trout *Oncorhynchus mykiss* (Walbaum, 1792). 240 fish in total were randomly assigned into four triplicate groups (20 fish/per aquarium) corresponding to four dietary treatments: control (PSO_0_; no addition of PSO), 0.5% (PSO_5_), 1.00% (PSO_10_), and 2.00% (PSO_20_) of PSO, respectively. After the 60 day-feeding trial, fish blood samples were collected and compared. Statistical analysis (one-way ANOVA) showed a significant (*P* < 0.05) effect of PSO on red blood cell count, hemoglobin concentration, mean corpuscular volume, mean corpuscular hemoglobin concentration, cholesterol, aspartate aminotransferase, alanine aminotransferase, and alkaline phosphatase parameters in PSO_5_ and PSO_10_ with regard to control. Moreover, a pronounced (*P* < 0.05) increased in weight gain, growth and feed conversion was found in fish fed with PSO supplemented diets. After the feeding trial, fish were challenged with *Y. ruckeri* and survival recorded for 20 days. Cumulative survival was 45.10% in fish fed with the control diet, whereas in fish fed with PSO_5_, PSO_10_, and PSO_20_ supplemented diets, survival was 58.82, 56.86, and 56.86%, respectively. In conclusion, dietary administration of PSO induced a reduction in mortality of rainbow trout infected with *Y. ruckeri*, intercalary significant differences occurred on growth performance and some blood values among treated groups. These positive effects of PSO could be considered for new applications in aquaculture.

## Introduction

In aquaculture diseases such as bacterial and viral infections are controlled using antibiotics and other chemotherapeutics that also promote the growth performance (Sasmal et al., 2005), and therefore, ensure the animal welfare, but some of these substances have deleterious effect on animals, fish, and humans. Some natural plant origin products like vegetables, herbs, spices, edible plants, and their extracts are not explained as traditional feed additives for animal nutrition. This plant derivates that act as growth promoters, immunostimulants or antimicrobial agents, represents viable and alternative to the use of antibiotics and other chemotherapeutics which are not harmful for environment and which have fewer side effect then chemical drugs (Galindo-Villegas and Hosokawa, 2004).

The effect of herbal products on different species of fish was studied in several previous researches (Alishahi et al., 2011; Tangestani et al., 2011; Asadi et al., 2012; Haghighi and Sharif Rohani, 2013).

Johnson and Banerji (2007) showed that herbal extract as an additive promotes hematological and biochemical performance, enhances the fish growth, and also protects from the diseases. However, studies related to pomegranate application in fish are scanty.

Pomegranate (*Punica granatum*) is an edible fruit belonging to the family of Punicaceae, native of Iran and widely cultivated in many countries. All anatomical parts of this fruit (seed, flower, juice, peel, leaf, bark, and root) possess antioxidant, anti-inflammatory, anti-cancer and anti-angiogenesis properties (Seeram et al., 2006). For this reason pomegranate has largely been used as a natural remedy against different pathological conditions (microbial infections, acidosis, dysentery, diarrhea, hemorrhage, and respiratory diseases) (Kim and Choi, 2009).

High levels of antioxidant are contained in pomegranate juice, peel, and seed oil (Schubert et al., 1999; Noda et al., 2002; Singh et al., 2002) and they represent potential candidates as a nutritional supplement for animals feed such as polyunsaturated oil called “punicic acid” (an omega 5 fatty acid) which has strong anti-inflammatory properties, for this it reason it is widely used in medicine and cosmetic (Wang et al., 2004; Boussetta et al., 2009).

In this research pomegranate seed oil (PSO), administered at different rates, was used as feed additive in diets of Rainbow Trout *Onchorhynchus mykiss*, that is a species farmed in aquaculture both for food and sports in Europe and one of the most valuable fish in world (Satari et al., 2007).

This study designed to determine the effects of PSO, which has not been investigated as a feed additives for rainbow trout on growth performance, hematological and biochemical values and resistance to *Yersinia ruckeri* infection.

## Materials and Methods

### Fish and Culture Conditions

A total of 240 cultured rainbow trout (6.79 ± 0.02 g) coming from a commercial aquaculture farm located in Turkey were allocated into 12 50 L aquarium (20 fish/per aquarium) and were allowed to acclimated for 15 days. Each fish was visually inspected externally according to United States Environmental Protection Agency (EPA) guidelines for qualitatively assessing fish health (Klemm et al., 1993).

Four experimental diets (PSO_0_, PSO_5_, PSO_10_, and PSO_20_) were obtained by adding the PSO decided by preliminary studies at a rate of 0, 0.5, 1.0, and 2.0% (**Table 1**).

**Table 1:** Percentage and proximate composition of the experimental diets containing supplement of different PSO rate.

Ingredients composition (g/100 g)	PSO_0_	PSO_5_	PSO_10_	PSO_20_
Fish meal^1^	40.2	40.2	40.2	40.2
Soybean meal^2^	30	30	30	30
Wheat meal^2^	7	7	7	7
Corn starch^2^	6.8	6.8	6.8	6.8
Fish oil^3^	12	11.5	11	10
Pomegranate oil	0	0.5	1	2
Vitamin-mineral^4^	4	4	4	4
Total	100	100	100	100
**Gross composition (% DM)**				
Protein	43.55	43.44	43.37	43.21
Lipid	17.21	17.29	17.37	17.40
Ash	7.66	7.71	7.56	7.64

*^*1*^Anchovy fish meal, Koptur Balıkçılık, Trabzon, Turkey.*

*^*2*^Soybean meal, Agromarin Yem San.ve Tic. A.Ş., İzmir, Turkey.*

*^*3*^Anchovy fish oil, AgromarinYem San.ve Tic. A.Ş., İzmir, Turkey.*

*^*4*^Vitamin mixture: vitamin A, 18000 IU/kg feed; vitamin D_*3*_, 2500 IU/kg feed; vitamin E, 250 mg/kg feed; vitamin K_*3*_, 12 mg/kg feed; vitamin B_*1*_, 25 mg/kg feed; vitamin B_*2*_, 50 mg/kg feed; vitamin B_*3*_, 270 mg/kg feed; vitamin B_*6*_, 20 mg/kg feed; vitamin B_*12*_, 0.06 mg/kg feed; vitamin C, 200 mg/kg feed; folic acid, 10 mg/kg feed; calcium D-pantothenate, 50 mg/kg feed; biotin, 1 mg/kg feed; inositol, 120 mg/kg feed; choline chloride, 2000 mg/kg feed.*

*Mineral mixture (mg/kg): Fe, 75.3 mg; Cu, 12.2 mg; Mn, 206 mg; Zn, 85 mg; I, 3 mg; Se, 0.350 mg; Co, 1 mg.*

Pomegranate seed oil was provided by “ONEVA,” a turkish factory. A basal diet was formulated using commercial feed ingredients (fish meal, soybean meal, wheat meal, corn starch, and fish oil). The ingredients were blended in a mixer and pelleted using meat grinder. Pellets were dried in a heater at 30°C for 48 h and stored at -18°C in plastic bags until use.

Pomegranate seed oil was added to the feed at 5, 10, and 20 g/kg. The control diet did not have any addition (**Table 1**).

The daily water change was made up to approximately half of total tank volume. The experiment diets were given for consumption to the fish three times a day as *ad libitum* at 09:00, 13:00, and 17:00 for 60 days. Water parameters were measured throughout the experiment as temperature was 15.2 ± 0.1°C, pH was 7.2 ± 0.2, and dissolved oxygen was 8.33 ± 0.2 mg/L.

### Determination of Growth Performance

When the 60 days experiment was over, each fish in all groups (a total of 240) were gained weight and the parameters of growth performance was calculated as following formulas:

$$\begin{array}{lll} Relative\;Growth\;Rate(RGR\;\%) & = & (final\;weight\;-\;initial\\ & & weight)/initial\;weight \end{array}$$

$$\begin{array}{lll} Specific\;Growth\;Rate(SGR) & = & 100\times \ln(final\;weight/initial\\ & & /days\;of\;experiment \end{array}$$

$$\begin{array}{lll} Feed\;Conservation\;Rate(FCR) & = & Feed\;Intake(g\;dry\;weight)/\\ & & (final\;weight\;-\;initial\;weight). \end{array}$$

The AOAC the standard method was applied for feed materials and experiment diets in order to determine moisture, ash, crude protein, and lipid (Helrich, 1990). The samples were digested with acid by using the auto Kjeldahl system and crude protein was detected according to Kjeldahl method. Soxtec system was used by the ether extraction method to determine crude lipid, moisture was determined using by oven drying at 105°C until constant weight was reached. The samples were placed in a muffle furnace at 550°C for 24 h and then ash content was measured.

### Blood Collection and Analyses

Blood was withdrawn from the caudal vein (three fish per aquarium per dietary group were anesthetised by MS222 as indicate by Topic Popovic et al. (2012), at the concentration of 0.7 g/L) at the end of the feeding experiment using 18G × 1.5 syringes rinsed with EDTA. Then blood was transferred into two different tubes, one (Miniplast 0.6 mL; LP Italiana Spa, Milan) containing ethylenediaminetetraacetic acid (EDTA) (ratio 1.26 mg/0.6 mL) as anticoagulant agent for the assessment of hematological parameters and the other (Terumo Corporation, Japan) without anticoagulant agent for the assessment of biochemical and immunological parameters.

Blood samples were centrifuged (10 min at 3000 *g* at 4°C) to obtained sera for biochemical and immunological analysis and stored at -20°C until they were used.

Serum lysozyme activity was assessed using turbidimetric analysis (Nudo and Catap, 2011). 25 μL of each serum were mixed with 175 μL of *Micrococcus luteus* (Sigma, ATCC 4698) suspension at 0.75 mg/mL in phosphate/citrate buffer. The mixture was incubated at 25°C, and its OD was measured after 30 min at 530 nm using a plate reader (Thermo Multiskan Go). Hen egg white lysozyme was used as an external standard. The rate of reduction was converted in absorbance of the samples to lysozyme concentration (μg/mL) using a standard curve.

Total myeloperoxidase (MPO) content was measured according to Quade and Roth (1997) and Sahoo et al. (2005) with minor modifications. 10 μl serum was diluted with 90 μl of HBSS without Ca^2+^ or Mg^2+^ in 96 well plate. 35 μL of 0.1 mg/mL 3,3′,5,5′-tetramethylbenzidine di-hydrochloride and 0.006% fresh hydrogen peroxide were added. After 2 min, 35 μL of 4 mol/l sulfuric acid was added to stop the reaction and the optical density was read at 450 nm in a plate reader.

The method of Blaxhall and Daisley (1973) was used to determine red blood cell (RBC) count (× 10^6^ per mm^3^), hematocrit (Hct; %), and hemoglobin (Hb) concentration (g/L). The RBC count was obtained with a Thoma hemocytometer using Dacie’s diluting fluid. A capillary Hct tube was used to determine the Hct value. The Hb concentration was measured by spectrophotometry (540 nm) via the cyanmethemoglobin method. Mean corpuscular volume (MCV), mean corpuscular Hb (MCH), and mean corpuscular Hb concentrations (MCHC) were calculated with the following formulae (Bain et al., 2006):

$$\begin{array}{r} MCV(\mu m^{3})\;=\;[(Hct,\;\%)\;\times \;10]/(RBC,\;\times \;10^{6}permm^{3})|\\ MCH(pg)\;=\;[(Hb,\;g/L)\;\times \;10]/(RBC,\;\times \;10^{6}permm^{3})\\ MCHC(\%)\;=\;[(Hb,\;g/L)\;\times \;100]/(Hct,\;\%). \end{array}$$

Sera bio-chemical variables [CHOL (cholesterol), TRIG (triglyceride), GLO (globulin), ALB (albumin), TP (total protein), and GLU (glucose)] were detected with bio analytic test kits (Bioanalytic Diagnostic Industry, Co.) and absorbance value was measured by using spectrophotometer (Optizen POP UV/VIS).

### Challenge Experiment With *Y. ruckeri*

A pathogenic pathogen *Y. ruckeri* (E42 Accession No: KX388238) was used. A stock of bacteria stored in tryptic soy broth containing 15% glycerol was prepared and maintained at -80°C. Briefly, 2 days before the challenge an aliquot of the bacteria was grown in tryptic soy broth medium at 22°C in a shaker incubator. After 1 day, the bacterial suspension was serially diluted in sterile PBS until the stock bacteria contained ∼10^7^ colony forming units (cfu)/mL.

For the challenge, 51 rainbow trout per groups (17 fish/aquarium) were anesthetised with MS222 (as described above), and injected intraperitoneally (i.p.) with 6.8 × 10^6^ cfu/mL of *Y. ruckeri* in PBS (0.1 mL/fish). The density of the bacteria was determined according to the previously calculated LD50 value for the rainbow trout juveniles. Dead fish were recorded daily and the fish were taken from the aquarium for 20 days. The bacterium was re-isolated from the dead fish.

### Procedure of Gas Chromatography–Mass Spectrometry (GC–MSD to Determine the Content of Pomegranate Oil

Analyses of the cold-press oil were performed using a Shimadzu GCMS QP 2010 ULTRA GC–MS system operating in the EI mode 0.70 kW, equipped with a split/splitless injector (250°C). The interface temperature was 250°C. Helium was used as carrier gas (1.26 mL/min) in capillary column and the capillary column used was: RTX-5MS (30 m; 0.25 mm; 0.25 μm). The temperature program was the same with that used for the GC analysis; split ratio 1:5. The injected volume was 1 μL. Acquisition mass range 45–450 m/z. The identification of the compounds was based on comparison of their retention times (RTs), and/or Wiley libraries, and the literature (Adams, 2007).

### Statistical Analysis

In this study, analytical data were represented as mean (M) ± standard error of the main (SEM) and they are the averages of three analyses carried out by the same operator. Samples exhibited parallel displacement to the standard curve. The overall intra-assay coefficient of variation was <9%.

Kolmogorov–Smirnov test was used to test for normality the data obtained for different blood parameters. The value *P* < 0.05 was considered statistically significant.

The influence of PSO on measured blood parameters in trout was evaluated by the application of One-way analysis of variance (ANOVA), Bonferroni’s multiple comparison test was used for *post hoc* comparison.

To analyze the data statistical software prism v. 5.00 (GraphPad Software, Ltd., United States, 2003) was used. Kaplan–Meier analysis was applied to estimate the survival of fish in each *Y. ruckeri*-challenged treatment group, and differences between groups were assessed with the log-rank (Mantel–Cox) test for pair wise comparisons.

## Results

Gas chromatography–mass spectrometry (GC–MS) analysis results showed that the major components of PSO were Squalene (45.09%), Δ- Tocopherol (37.01%), and ethyl linoleate (5.29%).

Variations (means ± SEM) of data related to growth performance, hematological, biochemical, and immunological parameters are reported in **Table 2**.

**Table 2:** Mean values ± SEM of growth, hematological, biochemical, and immunological parameters recorded in control and experimental groups.

Parameters	Control group		Experimental groups	
	PSO_0_	PSO_5_	PSO_10_	PSO_20_
**Growth**				
Initial weight (g)	6.82 ± 0.05^a^	6.76 ± 0.04^a^	6.78 ± 0.06^a^	6.81 ± 0.05^a^
Final weight (g)	18.16 ± 0.11^a^	20.56 ± 0.25^bc^	20.29 ± 0.22^bc^	16.60 ± 0.20^d^
RGR (%)	166.37 ± 5.01^b^	204.02 ± 3.12^a^	199.39 ± 3.07^a^	143.66 ± 5.51^c^
FCR	1.42 ± 0.02^b^	1.13 ± 0.05^c^	1.19 ± 0.01^c^	1.54 ± 0.03^a^
SGR	1.63 ± 0.03^b^	1.85 ± 0.01^a^	1.83 ± 0.02^a^	1.48 ± 0.04^c^
**Hematological**				
RBC (x10^6^ μL^-1^)	1.34 ± 0.06^a^	1.78 ± 0.05^bc^	1.88 ± 0.05^c^	1.50 ± 0.07^ab^
Hgb (mmol L^-1^)	2.25 ± 0.18^a^	3.51 ± 0.12^b^	3.35 ± 0.17^bc^	2.41 ± 0.09^a^
Hct (%)	29.67 ± 0.42^a^	31.83 ± 0.48^a^	31.67 ± 0.42^a^	31.33 ± 1.58^a^
MCV (fL)	222.70 ± 4.31^a^	180.00 ± 6.34^ab^	168.6 ± 5.50^b^	211.70 ± 18.01^ab^
MCH (pg/cell)	27.50 ± 2.88^a^	31.90 ± 1.25^a^	28.85 ± 2.00^a^	26.09 ± 1.52^a^
MCHC (g dL^-1^)	12.29 ± 1.05^a^	17.79 ± 0.76^b^	17.06 ± 0.81^bc^	12.51 ± 0.60^a^
**Biochemical**				
GLU (mmol L^-1^)	7.00 ± 0.60^a^	5.54 ± 0.65^a^	5.47 ± 0.55^a^	6.12 ± 0.42^a^
TP (g L^-1^)	33.70 ± 2.70^a^	35.1 ± 1.60^a^	35.90 ± 2.20^a^	35.60 ± 1.90^a^
ALB (g L^-1^)	6.00 ± 0.40^a^	5.50 ± 0.50^a^	5.60 ± 0.50^a^	5.00 ± 0.50^a^
GLO (g L^-1^)	27.60 ± 2.30^a^	29.60 ± 1.70^a^	30.30 ± 1.80^a^	30.60 ± 2.10^a^
CHOL (mmol L^-1^)	7.87 ± 0.39^a^	5.27 ± 0.05^b^	5.53 ± 0.16^b^	5.99 ± 0.22^b^
TRIG (mmol L^-1^)	1.35 ± 0.14^a^	1.82 ± 0.17^a^	1.67 ± 0.16^a^	1.31 ± 0.20^a^
AST (U/L)	180.40 ± 3.44^a^	131.00 ± 3.41^b^	145.20 ± 3.56^bc^	171.80 ± 3.28^a^
ALT (U/L)	23.87 ± 1.50^a^	15.30 ± 1.50^b^	14.76 ± 1.00^bc^	24.40 ± 1.27^a^
ALP (U/L)	63.17 ± 5.16^a^	31.97 ± 3.22^b^	29.57 ± 3.62^bc^	61.88 ± 3.57^a^
**Immunological**				
LYS (μg mL^-1^)	12.49 ± 1.24^a^	13.76 ± 1.08^a^	12.67 ± 1.24^a^	11.69 ± 0.90^a^
MPO (Abs 450 nm)	0.40 ± 0.08^a^	0.29 ± 0.09^a^	0.28 ± 0.09^a^	0.32 ± 0.06^a^

*Means with different alphabetical characters in the same row are statistically different (ANOVA, Bonferroni test; *P* < 0.05).*

*RGR, relative growth rate; FCR, feed conversion rate; SGR, specific growth rate; RBC, red blood cell; Hgb, hemoglobin; Hct, hematocrit; MCV, mean cell volume; MCH, mean cell hemoglobin; MCHC, mean cell hemoglobin concentration; GLU, glucose; TP, total protein; ALB, albumin; GLO, globulin; CHOL, cholesterol; TRIG, triglyceride; AST, aspartate transaminase; ALT, alanine transaminase ALP, alkaline phosphatase; LYS, lysozyme; MPO, myeloperoxidase.*

The application of one-way ANOVA showed a significant effect of PSO on some blood parameters in the experimental Groups with respect to control. In particular, groups PSO_5_ and PSO_10_ showed significantly (*P* < 0.05) higher levels of RBC, Hb, MCHC, and significantly (*P* < 0.05) lower levels of aspartate aminotransferase (AST), alanine aminotransferase (ALT), and alkaline phosphatase (ALP) respectively than control, while all three experimental Groups (PSO_5_, PSO_10_ and PSO_20_) showed significantly (*P* < 0.05) at lower levels of CHOL respectively than control. Only in group PSO_10_ significantly lower values of MCV with respect to control were showed.

No significant differences were found in Hct, MCH, GLU, TP, ALB, GLO, TRIG, LYS, and MPO between treatment groups.

**FIGURE 1:**
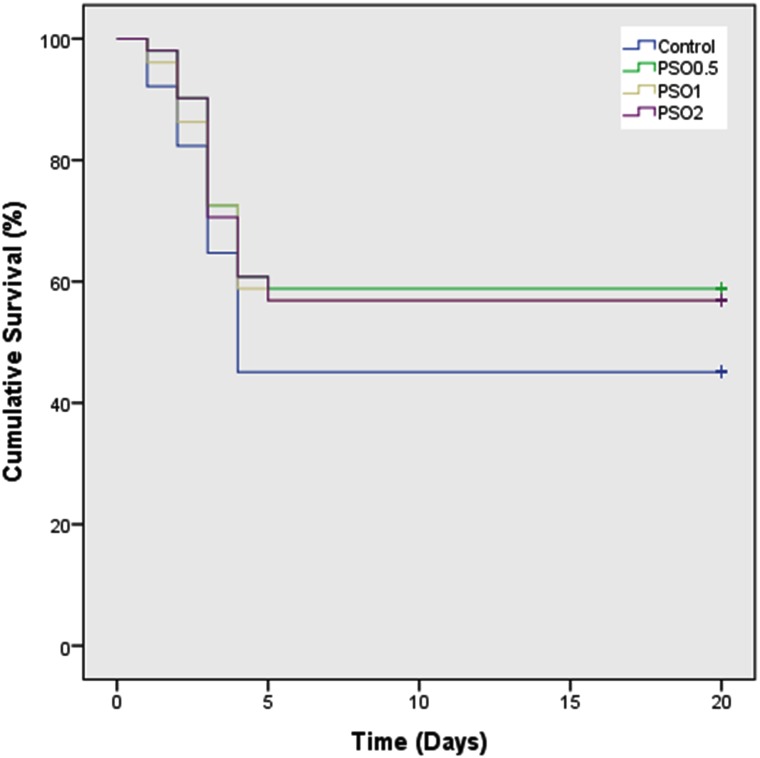
Kaplan–Meier survivorship curves (cumulative survival [%] over time [days]) for rainbow trout after challenge with *Yersinia ruckeri*; the fish were fed with pomegranate seeds oil supplemented diets (0, 5, 10, or 20 g of PSO/kg of feed; diets PSO_0_, PSO_5_, PSO_10_, and PSO_20_, respectively) prior to bacterial challenge 209 mm × 232 mm (300 × 300 DPI).

After 60 days of feeding, fish were challenged with *Y. ruckeri* and cumulative mortality was recorded for 20 days (**Figure 1**).

Cumulative survival was 45.10% in fish fed in the control diet. However, in fish fed the PSO_5_, PSO_10_, and PSO_20_ supplemented diets, survival was 58.82, 56.86, and 56.86%, respectively. All treated groups challenged with *Y. ruckeri* showed slightly reduced (*P* > 0.05) mortality compared to the PSO_0_.

## Discussion

Pomegranate seed oil has been established to have many pharmacological properties in terrestrial animal and aquatic species (Gil et al., 2000; Vidal et al., 2003; Badawi and Gomaa, 2016). But no information to date is available about its effects on growth and immunity of rainbow trout. The results of the present study demonstrated that PSO addition up to 10 g/kg in the diets barely affected the rainbow trout growth performance. Similar to our results the growth promoting effects has been reported other plant based feed additives (Nya and Austin, 2009; Nootash et al., 2013). In addition to this research (Baba et al., 2017) reported that Nile tilapia (*Oreochromis niloticus*) showed better growth performance when fed with 10 g/kg argan oil supplemented diets. This results suggesting that plant based feed additives has probable effects on modulation of intestine microbiota and digestive process therefore these supplements were improves growth performance of fish (MacLennan et al., 2002). Changes in RBC, Hct, Hb values, and erythrocyte indexes were important to evaluate the health status of organs (Başusta, 2005). In our study, hematocrit levels for rainbow trout fed with PSO supplemented did not show significant differences compared with the control group. Similar results were documented in different fish species fed with *Citrus sinensis* peel oil (Acar et al., 2015). Studies on fish hemoglobin shows that it is an important tool for health and it can be used for finding out about factors concerning their oxygen-carrying capacity (Wells et al., 2005). Our study showed that also RBC, Hb, and MCHC levels increased in fish treated with PSO at 0.5 and 1.00% levels. Hemoglobin (Hb) is a very important protein with which RBC is the key for blood oxygen (O_2_) transport in nearly all vertebrates and some invertebrates; this protein optimizes tissue O_2_ delivery by increasing the total O_2_ that can be transported in the blood (Rummer and Brauner, 2015). This suggests that PSO improves the performance of the oxygen transport thus promotes a better tissue perfusion.

Serum biochemistry represents a useful index of fish health status to determine physiological changes following different feeding experiment (Yılmaz and Ergun, 2012). The present study showed that dietary PSO showed no adverse effect on serum total protein, glucose, globulin, and albumin values of fish. However, the results of this study showed significantly decreased in cholesterol values of fish fed with PSO_5_ and PSO_10_ diets. The reduces in cholesterol values can be explained with the polyunsaturated fatty acids and other constituents of PSO. Baba et al. (2017) obtained similar results in tilapia (*Oreochromus niloticus*) fed with argan oil supplemented diets.

In particular, it was observed a significant reduction of AST, ALT, ALP (specific enzymes that are indicators of cellular toxicity) in fish treated at PSO_5_ and PSO_10c_ groups compared to control; instead at PSO_20_ these parameters returned control value. These results suggest an evident protective function or did not adversely effect of PSO on fish liver and are in line with those reported in a recent study by Badawi and Gomaa (2016) who studied the effects of diets supplemented by pomegranate peel extract (PPE) at rate of 0.1, 0.2, 0.3, and 0.5% in another species of fish (Nile Tilapia *O. niloticus*). A marked protective effect of PSO on liver function that decreased AST; ALT and ALP was also shown by Ibrahim (2010) in a previous study on rat. Decrease in enzyme activities can be regarded as an indicator of the protective effect of pomegranate oil on cells, tissues, and organs (Babalola et al., 2009) reported that vegetable oil sources (sunflower and cocoa butter) used in feeds of ductal fish increased liver enzymatic activities, which may be due to damage to liver cell membranes leading to the release of transaminases from the cytoplasm.

With regard to the immunological status, in a previous study of Badawi and Gomaa (2016), it was shown that PPE improved immune status by causing a significant increase in two important indices values of non-specific immunity (IgM and lysozyme) with respect to control; however, in our study the levels of lysozyme and myeloperoxidase did not show any significant difference (*P* < 0.05) in the fish fed with PSO supplemented diet with respect to control. The negligible effects on lysozyme and myeloperoxidase content showed in our results were also found in rainbow trout fed diets enrichment with herbal feed additives (Awad and Austin, 2010; Nya and Austin, 2011; Bulfon et al., 2017). Disagree with our results sweet orange (*Citrus sinensis*) essential oil (Acar et al., 2015) in tilapia seems to determine an increase of lysozyme activity. Similarly, in *Ictalurus punctatus* fed with oregano (*Origanum heraleoticum*) essential oil it was observed an increase in lysozyme activity respect to the control group (Zheng et al., 2009). This discrepancy is mainly due to the different substance administered, experimental conditions, fish species and probably at the different absorption capacities of the fish.

At the end of 60 days feeding trial to determine the resistance of rainbow trout to the enteric red mount disease fish were challenge with *Y. ruckeri*. The cumulative survival in rainbow trout fed with PSO_5_, PSO_10_, and PSO_20_ diets was 52.82, 56.86, and 56.86%, respectively and found significantly different from PSO_0_ group. It was observed that cinnamon (*Cinnamomum verum*) and clove oils (*Syzygium aromaticum*) reduced mortality in Nile tilapia after challenge with *S. iniae* and *Lactococcus garvieae*, respectively (Rattanachaikunsopon and Phumkhachorn, 2009, 2010). These results indicate that dietary treatment with various herb oils usually has a positive effect on infected fish, thus increasing survival rates. This aspect is probably due to the interdependent influences of the active components of herb oils and to the hormesis effect. Hormesis is a phenomenon typically associated with the fields of health and toxicology. “Hormesis is a dose-response relationship phenomenon characterized by low-dose stimulation and high-dose inhibition.” Depending on the nature of the parameter being affected, the direction of that response changes (Calabrese and Baldwin, 2003). Biological systems exposed to wide range of stimuli show diverse responses depending on the dose; hormesis is considered an adaptive function.

## Conclusion

The variation of responses in immunological parameters can be considered as driven variable condition of the animals but also it could be due to hormesis. The blood response to supplementation different levels of PSO could be hormetic, so in the future researches, it should be necessary to improve this research using other doses of PSO and different food times.

Results of our study showed that, PSO up to 10 g/kg in rainbow trout diets could determine an increase of growth performance and an improvement of innate immune response. The results indicated the potential of the PSO on *Y. ruckeri* infection and it should be as the potential use as a substitute for antibacterial to controlling disease in rainbow trout farming because as antibiotics it should be more future studies on the pharmaceutical properties on this seed using the trout as animal model.

## Ethics Statement

All the experimental procedures were carried out in accordance with the ethical considerations presented by the European legislation concerning the protection of animals used for scientific purposes (European Directive 2010/63). This study was approved by the local ethics committee for animal experiments of Çanakkale Onsekiz Mart University, Çanakkale, Turkey (Approval No. 2016/05-04).

## Author Contributions

ÜA, VP, and FF idea for this study. ÜA, OK, and SY designed the experiments. FF, CS, FA analyzed the data. VP, FF, GLP, and ÜA written the article. All authors have made substantial contributions to each step of the experimental procedure and article preparation.

## Conflict of Interest Statement

The authors declare that the research was conducted in the absence of any commercial or financial relationships that could be construed as a potential conflict of interest.
